# Palliative Radiation Therapy for Macroscopic Hematuria Caused by Urothelial Cancer

**DOI:** 10.1089/pmr.2020.0027

**Published:** 2020-09-29

**Authors:** Haiqin Zhang, Hidehiro Hojo, Vijay Parshuram Raturi, Naoki Nakamura, Masaki Nakamura, Masayuki Okumura, Yasuhiro Hirano, Atsushi Motegi, Shun-Ichiro Kageyama, Sadamoto Zenda, Tetsuo Akimoto

**Affiliations:** ^1^ Department of Oncology, Jinan Central Hospital, Cheeloo College of Medicine, Shandong University, Jinan, Shandong, P.R. China.; ^2^ Department of Oncology, Jinan Central Hospital Affiliated to Shandong First Medical University, Jinan, Shandong, P.R. China.; ^3^ Division of Radiation Oncology and Particle Therapy, National Cancer Center Hospital East, Kashiwa, Chiba, Japan.

**Keywords:** hematuria, palliative radiation therapy, urothelial cancer

## Abstract

***Background:*** To assess the efficacy and toxicity profiles of palliative radiation therapy (RT) for macroscopic hematuria (MH) caused by urothelial cancer.

***Methods:*** A total of 25 urothelial cancer patients with MH who underwent palliative RT between 2008 and 2018 were analyzed in this retrospective study. The hematuria-free survival (HFS) time was defined as the period from complete resolution of MH to the recurrence of MH, death, or the last follow-up examination. Adverse events were classified according to the Common Terminology Criteria for Adverse Events version 4.0.

***Results:*** By the end of the median follow-up duration of 90 days (11–886 days), complete resolution of MH had been achieved in 22 patients (88%), and the median interval between the start of RT and resolution of MH was 9 days (2–179 days). Of the 22 patients in whom the symptom resolved, 9 (41%) developed recurrent MH, and the median time to relapse of MH was 129 days (30–692 days). The median RT dose was 30 Gy (20–40 Gy). Nine (36%) patients received a blood transfusion before the RT. The three-month HFS rate was 52.1%. There was a significant difference in the three-month HFS rate between patients with and without a history of pretreatment blood transfusion (HFS rate: 34.6% vs. 61.5%, *p* = 0.03). Grade 2 urinary tract pain and grade 3 diarrhea were seen in one patient each.

***Conclusion:*** Palliative RT appeared to be effective with limited toxicities for urothelial cancer patients with MH.

## Introduction

Urothelial carcinoma (UC) is one of the most commonly occurring tumors in the world, especially in developed countries. Over half of the patients with UC show recurrence or tumor progression even after definitive treatments.^1^ In patients with UC, hematuria, especially macroscopic hematuria (MH), is the most common presenting symptom; it is often intractable, adversely affecting the quality of life of the patients. Uncontrollable MH is sometimes fatal. Several treatment approaches have been suggested, limited by poor treatment compliance or absence of proven effectiveness, including intravesical formalin treatment, alum irrigation, intravesical instillation of prostaglandin, hydrostatic bladder distention, urinary diversion, intra-arterial chemoperfusion with mitoxantrone, and embolization.^2^

Palliative radiotherapy (RT) is sometimes used as a treatment option for hematuria, with the reported hematuria improvement rate varying from 54% to 92%.^3–7^ A few reports of retrospective analyses of palliative RT for hematuria in patients with UCs have been published, and most are based on analyses of small cohorts. In 2016, a survey conducted by the palliative RT working group of the Japanese Radiation Oncology Study Group suggested that hemostatic irradiation is rarely performed at facilities in Japan and further that the number of fractions used varied significantly among facilities.^8^

Hence, the purpose of this study was to conduct a retrospective analysis of the efficacy and toxicity of palliative RT for MH in patients with urothelial cancer at our institution.

## Materials and Methods

### Ethics statement

This retrospective study was conducted with the approval of the ethics committee of our institution (approval number: 2017-440). The study was conducted in accordance with the Declaration of Helsinki.

### Patients

The study was conducted in 25 urothelial cancer patients with MH who received palliative RT between 2008 and 2018 at the National Cancer Center Hospital East. MH is defined as blood in the urine that can be seen with the naked eye. The patients were retrospectively enlisted from our database, and patients in whom the MH was caused by direct bleeding from the bladder tumor, which was established based on the clinical and/or diagnostic imaging findings, were analyzed. The clinical tumor stage (Union for International Cancer Control, eighth edition) was determined based on the findings of contrast-enhanced computed tomography and/or magnetic resonance imaging.

During the simulation, all patients were instructed to have an empty bladder and rectum. The clinical target volume (CTV) was the whole bladder, and in some patients, it included a part of the ureter, if the patient had been diagnosed as having ureteric invasion. The planning target volume was determined by the isotropic expansion of the CTV with a margin of 0.5–2 cm based on movement of the bladder in each patient. The four-field technique was adopted. Complete resolution of MH after RT was deemed as representing treatment success, while reappearance of MH was defined as relapse. Adverse events were classified according to the Common Terminology Criteria for Adverse Events version 4.0.

### Statistical analyses

EZR version 1.37 was used to perform all the statistical analyses.^9^ The hematuria-free survival (HFS) time was defined as the period from complete resolution of MH to the recurrence of MH, death, or the last follow-up examination. Chi-square test or Fisher's exact test was used to determine the significance of intergroup differences in discontinuous variables. The Kaplan–Meier method was used to evaluate survival probability. The survival between different groups was compared using the log-rank test. Univariate analysis and multivariate analysis were performed using the Cox regression model, with factors identified as significant by the former (*p* < 0.25) being entered into the latter model. The cutoff values of age, hemoglobin, and creatinine for analysis were set to the median values. The HFS in the multivariate analysis was performed using Akaike's information criterion (AIC) and Bayesian information criterion (BIC) methods. The Jonckheere–Terpstra test was performed to test for the trend in the period from the first day of treatment to the day of resolution of hematuria. *p* < 0.05 was considered being indicative of statistical significance. The variables used for the statistical analyses were age, gender, Eastern Cooperative Oncology Group (ECOG) performance status (PS), hemoglobin levels just before the radiotherapy, serum creatinine, the Union for International Cancer Control (UICC) tumor class, history of pretreatment blood transfusion, and history of chemotherapy before the palliative RT.

## Results

The characteristics of the 25 patients included in the analysis are shown in Table 1. The study population included 15 males and 10 females, with a median age of 73 years (range: 57–91 years); 16 patients (64%) had an ECOG PS of 0 to 1. The dominant histology was transitional cell carcinoma (22 patients; 88%), and the primary site of the tumor was the bladder in 21 patients (84%). Of the 25 patients, 24 were not suitable candidates for radical surgery because of advanced age (9; 36%), poor general condition (3; 12%), widespread metastasis (3; 12%), recurrence after transurethral resection of bladder tumor (7; 28%), or patient's refusal to undergo definitive treatment (3; 12%).

**Table 1.** Patient Characteristics

	No. of patients
Median age (range)	73 years (57–91)
Gender	
Male	15
Female	10
PS (ECOG)	
0–1	16
≥2	9
Histological type	
TCC	22
Adenocarcinoma	1
Squamous	1
NEC	1
Primary site	
Bladder	21
Ureter	3
Allantoic duct	1
UICC T classifications	
T2	10
T3	8
T4	7
Node classifications	
Negative	17
Positive	8
Distant metastases	
Yes	3
No	22
Hemoglobin (g/dL)	
<9.3	11
≥9.3	13
Unknown	1
Creatinine (mg/dL)	
<0.95	11
≥0.95	13
Unknown	1
Irradiation dose	
20 Gy/5 fr.	7
30 Gy/10 fr.	17
40 Gy/20 fr.	1
History of pretreatment blood transfusion	
Yes	9
No	16
History of chemotherapy before irradiation	
Yes	14
No	11

ECOG, Eastern Cooperative Oncology Group; fr, fractions; NEC, neuroendocrine carcinoma; PS, performance status; TCC, transitional cell carcinoma; UICC, Union for International Cancer Control.

Fourteen patients had a history of chemotherapy before irradiation. No patient whose interval between the last day of chemotherapy and the start of RT was less than 25 days was found (median duration 27 days; 25–237 days). After palliative RT, one patient underwent chemotherapy, one patient underwent reirradiation for recurrent hematuria, and one patient underwent chemotherapy followed by reirradiation. The remaining patients did not undergo any treatment intervention after palliative RT. The median hemoglobin level before palliative RT was 9.3 g/dL (range: 5.1–15.5 g/dL). The median hemoglobin level in the 9 (36%) patients who had received blood transfusion before the RT was 8.3 g/dL (range: 5.1–11 g/dL), while that in the remaining 16 (64%) patients was 10.6 g/dL (range: 6.9–15.5 g/L) (*p* = 0.02). All the patients received palliative RT alone, as follows: 20 Gy in 5 fractions in 7 (28%) patients, 30 Gy in 10 fractions in 17 (68%) patients, and 40 Gy in 20 fractions in 1 (4%). The fractionation schema was determined by the condition of the patients and/or the irradiated bladder volume. The median follow-up period after completion of RT was 90 days (range: 11–886 days).

Complete resolution of MH was achieved in 22 patients (88%). The median interval from the start of RT to complete resolution of MH was 9 days (range: 2–179 days). At two weeks after the start of RT, 15 (60%) patients showed no evidence of MH. Among the three patients who failed to show resolution of the MH, one died of multiple organ failure at the age of 88 years, after surviving for 13 days; the remaining two patients survived for 64 and 105 days, respectively. No significant intergroup differences in the rate of resolution of MH were found, including between those with and without pretreatment blood transfusion history (7/9 vs. 15/16, *p* = 0.53). Nine of the 22 (41%) patients who showed resolution of the MH developed recurrent MH, and the median period to the recurrence of MH was 129 days (range: 30–692 days). Among the nine patients with recurrent MH, two patients died within a week of recurrence due to severe anemia. The median survival duration of the nine patients was 278 days (range: 60–778 days).

**FIG. 1.**
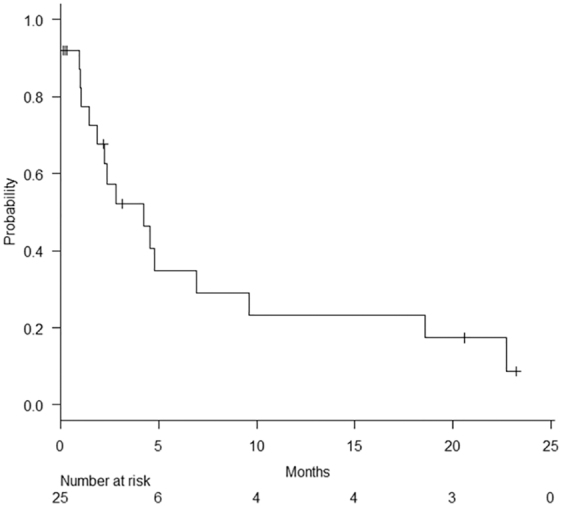
Hematuria-free survival.

**FIG. 2.**
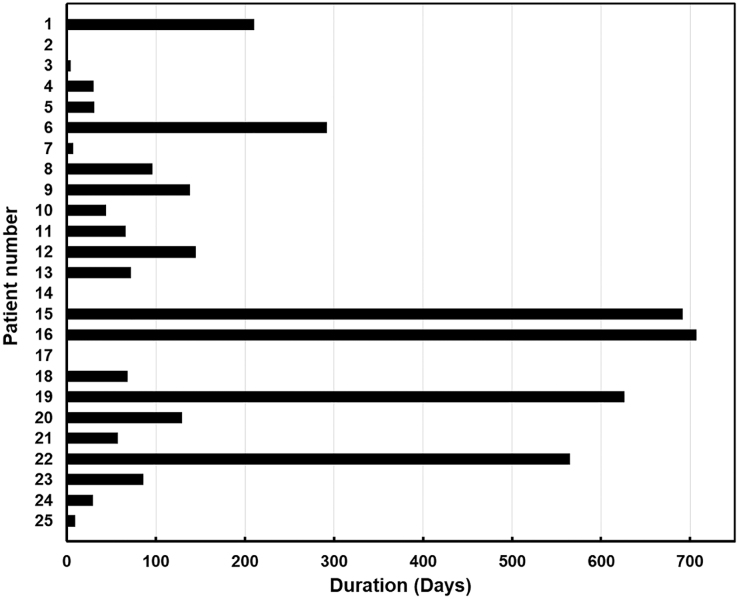
The duration of hematuria-free survival per patient.

**FIG. 3.**
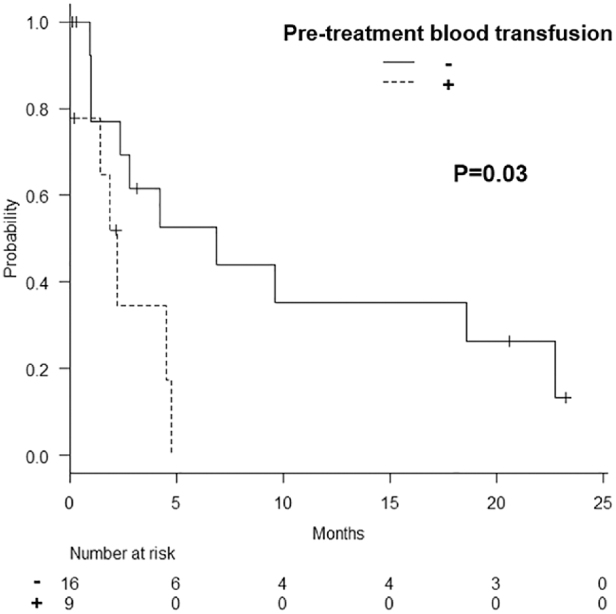
Hematuria-free survival by the history of pretreatment blood transfusion.

The HFS rate is shown in Figure 1, and the three-month HFS rate was 52.1%. The duration of HFS per patient is shown in Figure 2. There were significant differences in both the three-month HFS rate between patients with and without a history of pretreatment blood transfusion (HFS rate: 34.6% vs. 61.5%, *p* = 0.03) (Fig. 3). The results of the univariate and multivariate Cox regression analyses to identify factors influencing the HFS are shown in Table 2. Among these factors, patients with a history of blood transfusion before palliative RT had significantly worse HFS rates (*p* = 0.01). The period from the start of treatment to the day of resolution of MH was significantly longer in the patients with T3 and T4 disease than in those with T2 disease (median duration: 20 and 6 days, respectively; *p* = 0.01).

**Table 2.** Factors Related to Hematuria-Free Survival Using Cox Regression Analysis

Factors	HFS						
	Univariate (p)	Hazard ratio^a^	95% CI^a^	Multivariate (p)	Hazard ratio^b^	95% CI^b^	
Age (<73/≥73)	0.45	0.68	0.25–1.85				
Gender (male/female)	0.14	2.14	0.78–5.88	—	—	—	—
ECOG PS (0, 1/≥2)	0.13	0.31	0.07–1.40	—	—	—	—
Hb (<9.3/≥9.3 g/dL)	0.25	0.54	0.19–1.54				
Creatinine (<0.95/≥0.95 mg/dL)	0.76	0.86	0.32–2.30				
UICC T classifications (T1, 2/3, 4)	0.18	2.00	0.73–5.47	—	—	—	—
History of pretreatment blood transfusion	0.04	3.16	1.04–9.62	0.04	3.16	1.04	9.62
History of chemotherapy before irradiation	0.09	2.67	0.87–8.21	—	—	—	—
Radiation schedule (30 Gy in 10 fr. and more/20 Gy/in 5 fr.)	0.65	1.30	0.42–1.04				

^a^ Results from univariate analysis.

^b^ Results from multivariate analysis.

—, Variable not included in final step of multivariate analysis; CI, confidence interval; HFS, hematuria-free survival.

None of the patients developed grade 4 or grade 5 toxicities. The toxicities in the 25 patients included 1 (4%) patient with grade 1 urinary pain, 1 (4%) patient with grade 1 dermatitis, 3 (12%) patients with grade 1 diarrhea, and 4 (16%) patients with grade 1 increased urinary frequency. One patient each (4%) developed grade 2 urinary pain and grade 3 diarrhea.

## Discussion

The results of this retrospective single-institution study indicated that palliative RT may be an effective treatment method to manage MH caused by tumor recurrence or tumor progression, with acceptable toxicity.

Surgery is the standard treatment of choice for the management of advanced urothelial cancer,^10–14^ while definitive RT with or without chemotherapy has also been used as an alternative approach.^15–19^ However, if as per the tumor board discussion that the cure cannot be expected or the patients refuse the definitive treatment, for these patients, palliative therapy, RT, and palliative surgery are often performed for the purpose of symptom relief and supportive care.^5,20^

Palliative RT has good tolerance and good cost-performance characteristics.^21,22^ The main aim of palliative RT is to provide adequate symptomatic relief throughout a patient's anticipated life span. The efficacy of palliative RT for MH in patients with urological cancers has been reported in several retrospective studies.^3,4,6^ In regard to the mechanism of hemostasis induced by RT, platelet aggregation, injury of vascular endothelial cells, and induction of vascular embolization has been considered the main underlying mechanisms, in addition to tumor shrinkage.^23,24^ To obtain symptomatic relief or hemostasis, a high RT dose, as in definitive RT, is unnecessary. Only one randomized-controlled trial, MRC BA09,^5^ has been conducted until date to evaluate the efficacy and toxicity of palliative RT for symptomatic improvement in patients with bladder cancer who are deemed as unsuitable for curative treatment because of comorbidity. In this study, 500 patients from 20 centers received palliative RT at a dose of 35 Gy in 10 fractions or 21 Gy in 3 fractions. Symptomatic improvement was noted in ∼68% of patients. Among the patients suffering from hematuria, including microscopic hematuria, 88% were alleviated. The median time to deterioration of one or more bladder-related symptoms from the start of RT was nine months.

However, the optimal total radiation dose or fractionation schema has not been established, especially for palliative RT to treat MH, due to the limited clinical data available from retrospective studies. In a survey conducted in the Netherlands, nine distinct palliative RT schedules for bleeding tumors were identified, including 1 × 8 Gy, 2 × 8 Gy, 5 × 4 Gy, 5 × 5 Gy, and 10–13 × 3 Gy.^25^ In Japan, the fractionation schedules for palliative RT used to achieve resolution of MH varied widely according to the primary tumor sites, such as gastrointestinal and genitourinary tumors, and/or the patients' general condition. The most frequently used fractioned schema was 30 Gy administered in 10 fractions.^8^

In patients in a poor general condition with a limited survival prognosis, hypofractionated RT may be beneficial.^4,26^ Several studies have suggested that short-course RT was as efficient as RT administered in a higher number of fractions or long-course RT for obtaining bleeding control.^3,5,6,27^ In a previously conducted retrospective study, 20 Gy in 5 fractions (*n* = 46), 30 Gy in 10 fractions (*n* = 25), and 8 Gy in a single fraction (*n* = 21) were the most commonly used regimens in 112 patients who were receiving palliative RT for the control of MH occurring from tumors.^27^ Both longer RT regimens (>5 fractions) and shorter regimens (≤5 fractions) exerted equal hemostatic effect (*p* = 0.497) for an equal duration (*p* = 0.652). However, longer regimens caused frequent treatment interruptions and increased hospital days (22.2% vs. 5.3%, *p* = 0.020). Similarly, in the MRC BA09 study,^5^ there was no difference in the survival, symptomatic improvement rate, or toxicity between the two hypofractionated RT schedules (35 Gy in 10 fractions vs. 21 Gy in 3 fractions). Lacarriere et al.^6^ compared two RT schedules retrospectively: the standard treatment arm consisted of 30 Gy administered in 10 fractions over a period of two weeks, and the study treatment arm consisted of a hypofractionated regimen of 20 Gy administered in 5 fractions over a period of one week to patients with ECOG PS >2. No statistically significant difference was observed with respect to the MH control rate at two weeks after the start of treatment (54% vs. 79%, *p* = 0.139) or rate of relapse of MH at six months (62% vs. 71%).^4^ Recently, a retrospective study^3^ was conducted in 241 patients from 2 centers to investigate the efficacy of palliative RT in patients with bladder cancer, in which 5 different RT protocols were used: 8 Gy in 1 fraction (11%), 21 Gy in 3 fractions (15%), 20 Gy in 5 fractions (18%), 36 Gy in 6 fractions (36%), and 27.5 to 30 Gy in 8 to 10 fractions (18%). The results showed no significant difference in the clinical outcomes among the five different RT protocols.

In the current study, the resolution of MH was noted in 88% of all patients, and 60% of the patients showed no sign of MH after two weeks of palliative RT. The duration of hemostasis lasted for about four months. In addition, the toxicity of palliative RT was also acceptable; only one patient developed grade 3 diarrhea. Also, the results of the current study showed that the time to resolution of MH was longer in the patients with T3 or T4 disease compared with those with T2 disease. The majority of patients who developed recurrent hematuria were patients with T3 or T4 disease (7/9, 78%). This may indicate the possibility of the need for a higher dose of radiation in patients with more advanced or larger tumors.

Fourteen patients received chemotherapy before palliative RT and all the patients had a 25-day or longer interval before the start of RT. Thus, we considered that chemotherapy before irradiation was unlikely to be involved in hemostasis. Otherwise, two patients underwent chemotherapy after the palliative RT. Chemotherapy may have affected the lasting effect of hemostasis in the two treated patients.

Several limitations of this study must be pointed out, including the small sample size and short follow-up time. In addition, the information was obtained retrospectively from the medical record, and date when hemostasis stated is defined as the date of hemostasis. Therefore, it is possible that hemostasis was recorded long after the palliative RT. Therefore, further accumulation of patients is needed to arrive at a more concrete conclusion.

## Conclusions

Our study findings suggest that palliative RT could be an effective and safe treatment option to control macroscopic bleeding in patients with urothelial cancer. RT plays a pivotal role in the management of urothelial cancer, while palliative RT needs more attention. To date, only a handful of studies have shown the effects of palliative RT. Further research is required to determine the optimal individual treatment schedule, and select appropriate candidates for treatment, to guide clinical practice.

## Abbreviations Used

CTV: clinical target volume
ECOG: Eastern Cooperative Oncology Group
HFS: hematuria-free survival
MH: macroscopic hematuria
PS: performance status
PTV: planning target volume
RT: radiation therapy
UC: urothelial carcinoma
UICC: Union for International Cancer Control
